# Outpatient health care utilization and health expenditures of asylum seekers in Halle (Saale), Germany - an analysis of claims data

**DOI:** 10.1186/s12913-020-05811-4

**Published:** 2020-10-20

**Authors:** Andreas Niedermaier, Anna Freiberg, Daniel Tiller, Andreas Wienke, Amand Führer

**Affiliations:** 1 Institute of Medical Epidemiology, Biometrics and Informatics, Interdisciplinary Center for Health Sciences, Medical School of the Martin-Luther-University Halle-Wittenberg, Halle (Saale), Germany; 2 IT-Department, Data Integration Center, University Hospital Halle (Saale), Halle (Saale), Germany

**Keywords:** Secondary data analysis, Claims data, Asylum seekers, Health care utilization, Health care expenditures, Restricted access

## Abstract

**Background:**

Asylum seekers are a vulnerable group with special needs in health care due to their migration history and pre-, peri- and postmigratory social determinants of health. However, in Germany access to health care is restricted for asylum seekers by law and administrative regulations.

**Methods:**

Using claims data generated in the billing process of health care services provided to asylum seekers, we explore their utilization of health care services in the outpatient sector. We describe the utilization of outpatient specialties, prevalences of diagnoses, prescribed drugs and other health care services, as well as total costs of health care provision.

**Results:**

The estimated prevalence for visiting an ambulatory physician at least once per year was 67.5% [95%-Confidence-Interval (CI): 65.1–69.9%], with a notably higher prevalence for women than men. The diagnoses with the highest one-year prevalence were “Acute upper respiratory infections” (16.1% [14.5–18.0%]), “Abdominal and pelvic pain” (15.6% [13.9–17.4%]) and “Dorsalgia” (13.8% [12.2–15.5%]). A total of 21% of all prescriptions were for common pain killers. Women received more diagnoses across most diagnosis groups and prescribed drugs from all types than men. Less than half (45.3%) of all health care costs were generated in the outpatient sector.

**Conclusion:**

The analysis of claims data held in a municipal social services office is a novel approach to gain better insight into asylum seekers’ utilization of health services on an individual level. Compared to regularly insured patients, four characteristics in health care utilization by asylum seekers were identified: low utilization of ambulatory physicians; a gender gap in almost all services, with higher utilization by women; frequent prescription of pain killers; and a low proportion of overall health care costs generated in the outpatient sector. Further research is needed to describe structural and individual factors producing these anomalies.

## Introduction

### Background

Health care utilization is structured by health care needs on one hand and the availability of accessible, acceptable high quality health care services on the other [1]. In general, the health care needs of asylum seekers in Germany are very similar to those of the general population [2–4]. Nevertheless, due to upstream factors before, during and after migration [5, 6], asylum seekers are particularly vulnerable for a number of health risks [4, 5]. In consequence, there are some health care needs particular to asylum seekers [4, 7–10]: studies have shown higher prevalence of psychiatric disorders [11–13], communicable diseases [3, 14, 15], and problems of maternal health [12, 16].

Even though these special needs of asylum seekers are well established in the literature and the German health care system is able to provide acceptable, high quality care, with the *Asylum Seekers’ Benefits Act *(*“Asylbewerberleistungsgesetz”, ASBA*) German law creates barriers in access to health care [17]. The ASBA, passed into law in 1993, regulates the entitlement to health care for asylum seekers and people whose request for asylum was denied. The ASBA excludes this group from the statutory health insurance, appoints the municipal authorities with carrying health care costs and restricts entitlement to treatment to certain health care needs, namely, acute and painful conditions, as well as maternal care and certain preventative measures, e.g., vaccinations (§4) [17, 18]. Other treatments can be reimbursed if the social services office accepts written applications, arguing them as being essential in securing the patients’ health (§6) [17].

The restrictions in the ASBA are worded vaguely and are subject to the interpretation of the local social services office. The local authorities are relatively free in how to organize these provisions, and the practical implementation and approval practices differ greatly from district to district [19], which is why an asylum seeker’s chance of receiving adequate health care has been described as strongly depending on the chance of being distributed to a certain region in Germany [19, 20]. Some districts have chosen to task the statutory health insurance with organizing the cost reimbursement to the health care providers and have handed out electronic health insurance cards, equivalent to those of the statutory health insurance. In other districts, the municipal social services office hands out treatment vouchers on application for varying validity periods and differing coverage [19, 21]. The intricate process of voucher application and provision has to be passed by the asylum seeker in a time of illness, and it is as complicated by the asylum seekers’ frequent lack of knowledge about the legal framework and its implementation, lack of geographic mobility, and language barriers as the process of obtaining medical care afterwards. Therefore, it represents a significant barrier in access to health care for asylum seekers in itself [10, 19, 22]. In addition, the subsequent cost reimbursement process to the service providers creates wariness among physicians to treat asylum seekers as they fear not being reimbursed [19].

In addition, asylum seekers’ access to health services in Germany is complicated by modulating factors that are shared with other groups of migrants. Studies have identified limited geographic mobility [10, 23], language barriers [10, 23–25], lack of knowledge about the health care system for asylum seekers [10, 24], care providers’ lack of knowledge about their patients’ legal situation and realities of life [19, 24, 26] and discrimination and racism [24, 25, 27] as factors hindering asylum seekers in accessing adequate health care.

These restrictions and barriers impair the utilization of health services [2, 3, 19], alienate patients from the health care system [9, 10] and increase overall costs of health care provision to this group in the long term [18, 22, 28]. Nevertheless, little is known about the specifics of the utilization of health care by asylum seekers and the effects of these restrictions and barriers.

While the described structural factors determine the realization of access to health care, more individual factors also influence the interaction between the asylum seekers and the health system. Health literacy, as the individuals’ knowledge about health-related and health-seeking behaviour, may vary greatly among asylum seekers according to their educational background and origin country [29, 30]. Differing perceptions on the relations and roles of the asylum seeker and the care providers can lead to conflicts and hinder appropriate care [29, 31]. Conflicting perceptions on aetiologies and urgency of treatments have also been described to arise between patients and care providers from differing cultural backgrounds [10, 29, 31]. These “cultural factors” and their consequences for the clinical practise have been subject to debate [32–34].

Calls for monitoring of and data on health and health care utilization by this group have been voiced repeatedly [14, 35, 36] to improve the knowledge base and enable effective surveillance of the health status of this vulnerable group and the effects of policy decisions and interventions in health care for this population.

### The local context

Halle (Saale) is a medium-sized city with approximately 240,000 inhabitants, located in the east of Germany. Being an urban area, the density of medical facilities is high, and public transport is readily available, different from other more rural districts, where limited mobility has been described as a factor impairing asylum seekers’ utilization of health care [10, 23, 37]. To our knowledge, other structural mediating factors of access to health care do not differ much from other districts in Germany.

In Halle (Saale), as in most districts in Saxony-Anhalt, the social services office hands out health vouchers [10], but different from other districts, these are handed out unconditionally once every quarter, and they are valid until the end of the quarter [38]. With these vouchers the treating physicians have the promise that treatments covered in §4 ASBA will be paid for, but more extensive elective treatment options such as hospital treatments or therapeutic remedies (“Heilmittel”) have to be applied for in advance. Emergency treatments can be provided without prior application, but the care provider has to apply for cost reimbursement later. All these applications are then checked by the medically untrained staff of the social services office [10, 37]. If approved, the social services office reimburses the care providers after receiving the bills.

As the treatment vouchers handed out in Halle (Saale) have a longer period of validity than those handed out in other districts, and as they are handed out almost unconditionally, we consider it easier for asylum seekers from Halle (Saale) to access appropriate health care here than in most other districts that have implemented the voucher model. Yet the social services office still functions as a gate keeper compared to other districts that have tasked the statutory health insurance with handing out electronic health insurance cards to asylum seekers, facilitating access to adequate health care considerably as no application for a voucher is necessary in a time of illness.

### Aims

With our study we want to answer the calls for contributions to the knowledge base about asylum seekers’ health care utilization. This explorative study aims to describe the outpatient health care utilization by asylum seekers in Halle (Saale) and the total health expenditures for this population. By analysing claims data generated in the billing process of health services held by the municipal social services offices, we highlight the unique potential of this data source to monitor health care utilization by asylum seekers. Drawing on other sources of utilization data, we try to identify anomalies and generate hypotheses that warrant further research.

Empirical data on health care utilization by asylum seekers is essential to improve provision processes and health outcomes. With our study, we want to support policy makers and health care professionals in facilitating equitable access to health care for asylum seekers by contributing to a knowledge base about asylum seekers’ health care utilization.

## Methods

This retrospective study uses claims data of the social services office in Halle (Saale), Germany, to describe asylum seekers’ utilization of health services in the year 2015. We analyse the contacts between asylum seekers and the health system, i.e., visited specialties, diagnosed morbidity, services provided such as prescribed medication or other treatments, and costs thereof. With this population-based data set, we can show frequencies, prevalences and other key characteristics of the utilization of health care on an individual level unrestrained by selection or recall bias.

### Study population

Halle (Saale), Germany was chosen as a study site because of the pre-existing cooperation between our research group and the social services office that enabled access to the data. All asylum seekers registered with the social services office of Halle (Saale), Germany, and therefore entitled to provisions under the ASBA at any time in 2015 for at least 1 day were included in this analysis, whether they had received any medical services or not.

### Data source

Because of the aforementioned organization of the provision and payment of health care for asylum seekers through the municipal social services offices, these offices hold not only the demographic data of each entitled asylum seeker but also the complete billing documents of all health care that is provided to this population. The bills are stored in the social services office in paper form. The information of the paper-based bills was entered into a MySQL-database through a custom-made web-based data entry form. The data were anonymized in the process of digitalization. Data cleansing and analyses were performed using SAS/STAT® 9.4 [39].

To quantify the error rate of typing in the data, 495 bills of ambulatory physicians with 96 variables of mixed types (dates, open-text, continuous) each were randomly selected for double data entry. A comparison of the two sets of data revealed an error rate of 0.35% on a per-variable basis, which was lower than comparable results from the literature [40].

### Variables

For each individual matching the above-mentioned inclusion criteria, the social services office provided information on gender, date of birth, country of origin and first and, where available, last day of entitlement to services under the ASBA, from which we calculated time under observation in our study as days of entitlement in the year 2015.

Each recorded billing document contained information on the first date of contact with the billing doctor’s office or hospital, the name and specialization of the billing physician or hospital, procedure codes classified through the standardized classification manuals for ambulatory physicians (EBM) and dentists (BEMA), respectively [41, 42], diagnoses classified through ICD-10 [43], PZN-Codes for prescribed pharmaceuticals [44], other medical services described in text, and the costs that were billed with the social services office. Data in prescription documents of therapeutic remedies and medical aid products were recorded as classified in the statutory manuals *catalogue of non-physician care* (“Heilmittelkatalog”) [45] and *catalogue of medical aid products* (“Hilfsmittelkatalog”) [46], respectively.

Outpatient services are billed per case. A case is generated by at least one visit of the patient to one doctor or clinic in a quarter and contains all contacts and services provided during that quarter. Exempted from this rule are laboratory physicians, who are consulted by all other specialties for diagnostic tests and bill each set of diagnostic tests separately. Therefore, one patient can generate up to four cases with one ambulatory physician in one year, but a virtually unlimited number of cases with laboratory physicians. Physicians’ specialties were taken from the identifying number unique to each ambulatory physician with the last two digits describing the physicians’ specialty [47]. This number is noted on all prescriptions. On bills from ambulatory offices, this number was not available. For doctors’ offices with more than one specialty, we derived the specialty from specialty-specific procedure codes in the EBM. For analysis, we grouped general practitioners, family doctors and internists who work as family doctors under the label “family doctors”. In Germany, obstetrics and gynaecology is practised by a single specialty and are therefore not being differentiated here.

All specialties have to state legitimating diagnoses in their bills, except for laboratories and diagnostic radiologists. In the analysis of diagnoses, we counted how many patients received a unique diagnosis at least once in the whole year, discounting multiple diagnoses of the same disease and regardless of which physician made the diagnosis, as we could not differentiate if a diagnosis was made for multiple accounts of one illness or if it was ongoing. Physicians are required to qualify the diagnoses as either affirmed (G), suspected (V), ruled out (A) or “symptom free state after diagnosis” (Z). Except when stated otherwise, diagnosis codes were excluded that were qualified as a ruling out of this disease. Thus, we counted only reports of a suspected or affirmed diagnosis or of a symptom-free state following a diagnosis.

For pharmaceuticals, PZN-Codes that describe unique sold units were transcoded to the Anatomical Therapeutic Chemical (ATC) Classification System [48], which describes active ingredients grouped by area of therapeutic use. For analysis, the fifth level was used for individual drugs and the second level for therapeutic subgroups. In this article, we took from inpatient bills only the billed costs and reason for admission, which qualifies emergency or regular cases [49].

### Statistical procedures

For each analysis, we first provide descriptive statistics to show the crude absolute and relative frequencies of diagnoses and services. Second, we show rates per person-year to account for the vastly differing times of observation, as individual periods of entitlement do overlap with our study period differently. As the distributions of counted events among individuals were highly skewed, we then show percentages of the population having received a certain service or diagnosis at least once over the course of one year of observation. These percentages or one-year estimates are calculated by using Kaplan-Meier analysis to account for shorter observation times (See Supplement 2). These estimates represent administrative one-year prevalences for the underlying population for each diagnosis or service and are labelled as such.

### Ethics approval

This secondary data analysis uses administrative data that fulfils all necessary requirements of the Federal data protection act of the Federal Republic of Germany. As this study only uses anonymized secondary data, according to national guidelines, no clearance by the ethics committee was necessary [50].

## Results

### Demography

In total, 4107 asylum seekers were included in the study (men: *n* = 3004, 73.1%; women: *n* = 1103, 26.9%). People originated from a total of 67 countries, most of them from Syria (*n* = 1957, 47.7%), Afghanistan (*n* = 354, 8.6%), Iran (*n* = 180, 4.4%), Somalia (*n* = 173, 4.2%) and Benin (*n* = 168, 4.1%). A total of 37 people were of unclear origin, and seven were stateless. The median time under observation during the year 2015 was 106 days (min: 1; max: 365). All 4107 people in sum contributed 1786.6 person-years (PY; men: 1307.5PY; women: 475.7PY) during the year 2015. Gender distribution was roughly even in age groups below 15 and above 45. Between age 15 to 45 years, men were overrepresented. More details on the demographic composition are given in Table 1.

**Table 1:** Demographic characteristics of the study population

	Male		Female		All	
	n	%	n	%	n	%
**Age**						
**0- < 5 yrs**	154	3.75	158	3.85	312	7.6
**5- < 10 yrs**	118	2.87	119	2.9	237	5.77
**10- < 15 yrs**	110	2.68	64	1.56	174	4.24
**15- < 20 yrs**	344	8.38	92	2.24	436	10.62
**20- < 25 yrs**	655	15.95	134	3.26	789	19.21
**25- < 30 yrs**	601	14.63	164	3.99	765	18.63
**30- < 35 yrs**	424	10.32	124	3.02	548	13.34
**35- < 40 yrs**	249	6.06	95	2.31	344	8.38
**40- < 45 yrs**	154	3.75	48	1.17	202	4.92
**45- < 50 yrs**	113	2.75	32	0.78	145	3.53
**50- < 55 yrs**	42	1.02	25	0.61	67	1.63
**55- < 60 yrs**	17	0.41	20	0.49	37	0.9
**60- < 65 yrs**	9	0.22	11	0.27	20	0.49
**> 65 yrs**	14	0.34	17	0.41	31	0.75
**Sum**	3004	73.14	1103	26.86	4107	100
**Country of origin**						
**Syria**	1461	35.57	496	12.08	1957	47.65
**Afghanistan**	244	5.94	110	2.68	354	8.62
**Iran**	115	2.8	65	1.58	180	4.38
**Somalia**	109	2.65	64	1.56	173	4.21
**Benin**	141	3.43	27	0.66	168	4.09
**India**	88	2.14	33	0.8	121	2.95
**Guinea-Bissau**	101	2.46	15	0.37	116	2.82
**Niger**	94	2.29	7	0.17	101	2.46
**Russian Federation**	49	1.19	49	1.19	98	2.39
**Iraq**	61	1.49	35	0.85	96	2.34
**Unknown/missing**	26	0.63	11	0.27	37	0.9
**Others**	515	12.54	191	4.65	706	17.19
**Sum**	3004	73.14	1103	26.86	4107	100
**Time under observation**						
**Less than 30 days**	249	6.06	125	3.04	374	9.11
**31-60 days**	591	14.39	300	7.3	891	21.69
**61–90 days**	375	9.13	139	3.38	514	12.52
**91–120 days**	467	11.37	103	2.51	570	13.88
**121–180 days**	360	8.77	45	1.1	405	9.86
**181–240 days**	137	3.34	47	1.14	184	4.48
**241–300 days**	132	3.21	47	1.14	179	4.36
**More than 300 days**	693	16.87	297	7.23	990	24.11
**Sum**	3004	73.14	1103	26.84	4107	100

Due to changes in the demography of people coming to Germany as asylum seekers during 2015, the study population’s demography also changed in the course of the year. Most notably, the population grew from 1301 people with entitlement on January 1, 2015 to 3134 people on December 31, 2015. The percentage of Syrian nationals increased from 15.9% (January 1, 2015) to 47.2% (December 31, 2015).

### Frequency of outpatient care

**Fig. 1:**
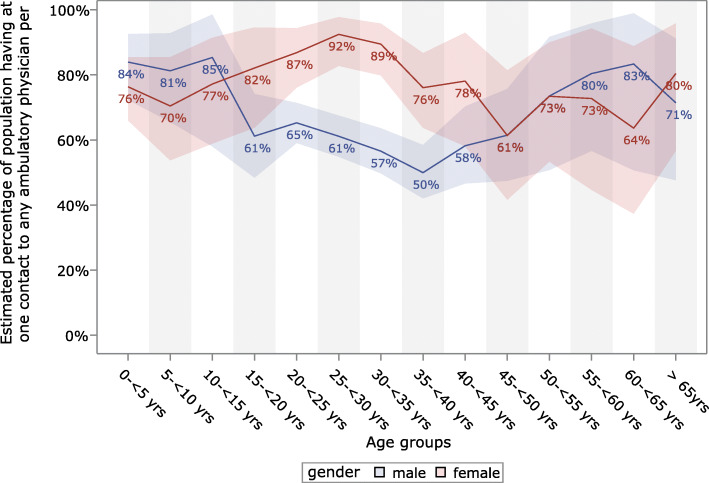
Estimated one-year prevalence of having at least one contact with any outpatient physician

We recorded a total of 7809 billed cases from ambulatory physicians, 4555 for men and 3254 for women. This amounted to a rate of 437.9 ambulatory cases per 100 person-years (men: 348.4 cases/100PY; women: 684.0 cases/100PY) across all specialties, including visits to emergency departments. These cases were not evenly generated by all individuals. Only 46.5% of asylum seekers visited an ambulatory physician. After correction for shorter observation times through Kaplan-Meier analysis, the one-year prevalence of visiting an ambulatory physician at least once was 67.5% [65.1–69.9%]. This prevalence differs notably between age groups and gender: while 81.8% [77.9–85.5%] of women were estimated to have at least one contact with an ambulatory physician, only 62.5% [59.6–65.4%] of men had at least one contact. Similarly, 92.4% [82.7–97.8%] of all women between 25 and 30 years of age were estimated to have at least one visit to a physician, while middle-aged men (age 35 to 40) had the lowest prevalence (50.0% [42.0–58.5%]). Figure 1 shows the estimates of the age-related prevalence of people having at least one contact with an outpatient health care provider per year.

The distribution of case numbers generated by individual asylum seekers was highly skewed. While many asylum seekers did not generate a single case in one year of observation (32.5% [30.1–35.0%], very few (2.8% [1.9–3.9%]) had 20 or more cases.

**Fig. 2:**
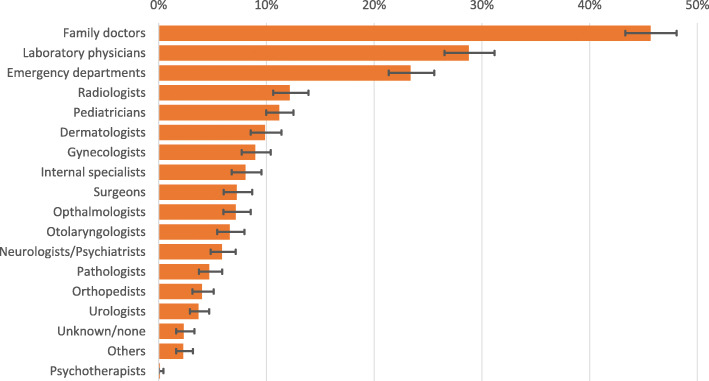
Most commonly consulted ambulatory specialties with estimated one-year prevalence having at least one contact

Family doctors are the most consulted specialty. The one-year prevalence of people having at least one contact with a family doctor was 45.7% [43.3–48.1%]. Next are laboratory physicians (28.8% [26.5–21.2%]) and emergency departments (23.4% [21.4–25.6%]). We estimated that 65.0% [58.6–71.2%] of all patients younger than 18 years consulted a paediatrician at least once in one year, and 33.3% [29.1–38.0%] of all women of any age consulted a gynaecologist. Less than 1% (0.13% [0.04–0.44%]) of all asylum seekers visited a psychotherapist. Figure 2 shows the estimates of the one-year prevalences of having at least one contact to the most commonly consulted ambulatory specialties.

### Diagnoses in outpatient care

Of a total number of 17,100 ICD-10-coded diagnoses, 89.1% were marked as affirmed, 6.6% as suspected, 2.3% as ruled out and 1.4% as symptom-free. For 109 diagnosis codes (0.6%), this information was missing or invalid. In the following analyses, we excluded all diagnoses that were marked as ruled out.

The distribution of diagnosis frequencies per person was heavily skewed. While 64.1% of all persons of our study population were not once diagnosed by any ambulatory physician, 1.5% of all people received more than 20 unique diagnoses (Median: 0; third quartile: 3; maximum: 44).

The diagnosis with the highest one-year prevalence was “J06: Acute upper respiratory infections”: 16.1% [14.5–18.0%] of all asylum seekers received this diagnosis at least once per year. Next were conditions that are also common in the general German population [51, 52]: “R10: Abdominal and pelvic pain” (15.6% [13.9–17.4%]) and “M54: Dorsalgia” (13.8% [12.2–15.6%]). The two psychiatric disorder groups “F32: Depressive episode” and “F43: Reactions to severe stress and adjustment disorders” were also diagnosed frequently with a one-year prevalence of 5.5% [4.5–6.7%] and 4.0% [3.2–5.0%]. In one year 2.8% [2.1–3.7%] of our study population was estimated to receive at least one diagnosis of the group of codes that describe Tuberculosis (A15–19). The estimated one-year prevalence of a diagnosis relating to of HIV (B20–24, U60, Z21) was 0.4% [0.2–0.8%]. Supplement 3 shows more detailed information on single diagnosis codes and diagnosis groups.

Analysis of chapters of the ICD-10 reflects the analysis of singular diagnosis codes. Table 2 shows estimated one-year prevalences of people having at least one diagnosis from each chapter, stratified for gender. The prevalences were notably higher for women than men in every chapter but one, with the largest difference in chapters “14: Diseases of the urinary system” and “3: Diseases of the blood, blood forming organs and the immune mechanism”. More men only were diagnosed with chapter “19: Injury, poisoning and certain other consequences of external causes“.

**Table 2:** One-year prevalence of diagnoses by chapters of the ICD-10 (with 95% confidence intervals)

Diagnosis groups	One-year prevalence		
Chapter ICD-10	% of men [95%-CI]	% of women [95%-CI]	% of all [95%-CI]
1: Certain infectious and parasitic diseases	19.1 [17.0–21.6]	28.0 [23.9–32.6]	21.6 [19.6–23.7]
2: Neoplasms	2.6 [1.9–3.6]	10.9 [8.4–14.1]	4.9 [4.0–6.0]
3: Diseases of the blood, blood-forming organs and certain disorders inv. the immune mechanism	2.2 [1.5–3.2]	12.0 [9.4–15.4]	4.8 [3.9–6.0]
4: Endocrine, nutritional and metabolic diseases	5.4 [4.2–6.8]	17.9 [14.6–21.8]	8.7 [7.5–10.2]
5: Mental and behavioural disorders	11.8 [10.1–13.7]	21.1 [17.6–25.1]	14.2 [12.7–16.0]
6: Diseases of the nervous system	7.2 [5.9–8.8]	10.8 [8.3–14.0]	8.1 [6.9–9.6]
7: Diseases of the eye and adnexa	9.8 [8.2–11.6]	14.2 [11.2–17.9]	11.0 [9.6–12.7]
8: Diseases of the ear and mastoid process	4.4 [3.4–5.7]	8.8 [6.4–11.9]	5.6 [4.6–6.9]
9: Diseases of the circulatory system	5.2 [4.2–6.6]	12.0 [9.2–15.4]	7.1 [6.0–8.5]
10: Diseases of the respiratory system	24.5 [22.2–27.1]	36.1 [31.9–40.8]	27.6 [25.5–29.8]
11: Diseases of the digestive system	16.9 [14.8–19.3]	24.2 [20.6–28.4]	18.8 [17.0–20.8]
12: Diseases of the skin and subcutaneous tissue	14.9 [13.1–17.0]	25.3 [21.5–29.7]	17.7 [15.9–19.6]
13: Diseases of the musculoskeletal system and connective tissue	21.0 [18.8–23.5]	24.8 [21.2–29.0]	22.0 [20.0–24.0]
14: Diseases of the genitourinary system	7.7 [6.3–9.3]	39.9 [35.5–44.6]	16.0 [14.3–17.8]
15: Pregnancy, childbirth and the puerperium	–	15.9 [13.0–19.5]	4.2 [3.4–5.3]
16: Certain conditions originating in the perinatal period	0.3 [0.1–0.5]	0.4 [0.1–1.1]	0.3 [0.2–0.5]
17: Congenital malformations, deformations and chromosomal abnormalities	2.2 [1.5–3.3]	4.7 [3.1–7.0]	2.9 [2.2–3.8]
18: Symptoms, signs and abnormal clinical and laboratory findings, not elsewhere classified	27.9 [25.4–30.6]	54.6 [49.8–59.5]	34.9 [32.5–37.3]
19: Injury, poisoning and certain other consequences of external causes	16.6 [14.5–18.9]	13.0 [10.1–16.5]	15.6 [13.9–17.5]
20: External causes of morbidity and mortality	0.3 [0.1–0.9]	0.3 [0.0–2.3]	0.3 [0.1–0.8]
21: Factors influencing health status and contact with health services	12.2 [10.5–14.2]	52.4 [47.6–57.4]	22.7 [20.7–24.8]
22: Codes for special purposes	6.8 [5.5–8.5]	15.0 [12.1–18.6]	8.9 [7.6–10.3]

### Prescriptions

The 5346 analysed prescriptions contained a total of 7989 prescribed drugs. The identifying ATC-Code was missing or invalid in 1.85% (*n* = 146) of all data entries. A total of 1485 (36.1%) patients were prescribed at least one drug. The estimated prevalence of receiving at least one prescribed drug per year was 57.2% [54.7–59.7%] and was considerably higher for women (70.0% [65.5–74.4%]) than for men (52.6% [49.7–55.6%]). A total of 1.3% [1.2–1.5%] of all asylum-seekers were estimated to receive more than 20 prescriptions in one year. One person received a maximum of 75 prescribed drugs. Per 100 person-years, this amounted to 448.0 prescribed drugs, with 366.2 prescribed drugs per 100 PY for men and 672.9 prescribed drugs per 100 PY for women.

The most frequently prescribed drug is Ibuprofen, with 14.1% of all prescriptions. More than one third of all people were estimated to receive at least one prescription of Ibuprofen in one year. Four of the seven most frequently prescribed drugs are also mostly prescribed as painkillers (Ibuprofen, Metamizole, Paracetamol, Diclofenac), together making up 21% of all prescriptions. Third ranks Xylometazoline, the active ingredient of nasal decongestants that is almost exclusively prescribed for children (97% of all prescriptions of Xylometazoline were for people of age < 15). The same is true for Hederae helicis folium, ranked 8th, which is the active ingredient of expectorant syrups. Mirtazapine ranks 6th, being a commonly prescribed antidepressant indicated for episodes of major depression. Supplement 4 shows the Top 10 most prescribed drugs with percentages of all prescriptions and one-year prevalences of people receiving at least one prescription per person-year stratified by gender.

The findings for therapeutic subgroups reflect the frequencies of single drugs reported above. Antibacterials for systemic use are ranked second in this analysis, with an estimated 18.3% [16.5–20.3%] of all people treated at least once per year, but do not show up in the single drug analysis because of the multitude of subgroups and single drugs making up this group. The antiinflammatory and antirheumatic products (M01) prescribed here are mostly (88.8%) Ibuprofen (M01AE01) and psychoanaleptics (N06) prescribed here are exclusively antidepressants (N06A). Surprisingly, antimycobacterials (J07) rank 9th in frequency, with 3.1% of all prescriptions and 1.5% [1.0–2.2%] of all people being treated at least once in one year. Table 3 shows the most commonly prescribed drug groups with with percentages of all prescriptions and estimated one-year prevalences of receiving at least one prescription.

**Table 3:** Most commonly prescribed drug groups with percentages of all prescriptions and estimated one-year prevalences of receiving at least one prescription

Prescribed drugs		Proportion	One-year prevalence		
Code	Group name	% of all prescriptions	% of men [95%-CI]	% of women [95%-CI]	% of all [95%-CI]
M01	Anti-inflammatory and antirheumatic products	15.9	33.2 [30.5–36.1]	41.1 [36.5–46.0]	35.3 [33.0–37.8]
J01	Antibacterials for systemic use	7.0	14.6 [12.7–16.7]	28.5 [24.5–33.1]	18.3 [16.5–20.3]
N02	Analgesics	6.3	14.0 [12.1–16.1]	25.0 [21.1–29.5]	17.1 [15.3–19.1]
A02	Drugs for acid related disorders	6.3	14.6 [12.6–16.8]	16.2 [13.2–19.8]	14.9 [13.3–16.7]
R05	Cough and cold preparations	5.7	7.2 [6.0–8.7]	14.2 [11.3–17.7]	9.1 [7.9–10.5]
R01	Nasal preparations	5.6	7.2 [5.9–8.9]	16.2 [13.2–19.8]	9.6 [8.3–11.1]
N06	Psychoanaleptics	4.6	3.8 [2.9–5.0]	7.8 [5.6–10.8]	4.9 [3.9–6.1]
N05	Psycholeptics	4.5	2.6 [1.8–3.6]	6.3 [4.3–9.2]	3.6 [2.8–4.7]
J04	Antimycobacterials	3.1	1.6 [1.0–2.5]	1.2 [0.6–2.5]	1.5 [1.0–2.2]
D07	Corticosteroids, dermatological preparations	3.1	7.0 [5.7–8.7]	10.8 [8.1–14.2]	8.1 [6.8–9.5]
J07	Vaccines	2.6	3.8 [2.8–5.2]	8.4 [6.0–11.7]	5.1 [4.1–6.4)

### Others

#### Dentists

Our data contained 708 bills for dentist cases, amounting to 39.7 cases (males: 37.3; females: 46.2) per 100 person-years. These cases contained a total of 5063 reported procedure codes (classified through the BEMA [42]). Per case a mean of 7.1 procedure codes was reported (median: 5; Min 0: Max: 61). The most common procedure code was “Ä1: Consultation of a patient, even by telephone”, with 17.2% of all reported codes; 98.1% of all cases contained this code. Second in frequency was “40: Infiltration anaesthesia”, with 8.1% of all codes, and 38.8% of all cases containing this code, and “32: Preparation of a root canal, per canal”, with 7.3% of all reported codes, and 21.6% of all cases containing this code. Looking into subgroups of the BEMA, the most common subgroups were “101: Diagnostics and consultations”, with 99.0% of all cases containing procedure codes of this subgroup, “109: Anaesthesia”, reported in 55.9% of cases, and “102: X-ray radiography”, reported in 52.5% of all cases. A total of 22.7% of all cases contained only diagnostic procedure codes (Subgroups “101”,“102”). No cases contained prophylactic procedure codes (“103: Prophylactic procedures”). The estimated one-year prevalence of visiting a dentist at least once was 24.0% [22.0–26.1%].

#### Therapeutic remedies

A total of 188 prescriptions for therapeutic remedies were counted. Per 100 person-years 10.5 prescriptions of this kind were billed. Overall, 86.7% of these concerned physiotherapy, 8% occupational therapy, 3.2% speech therapy, and 2.2% others/unknown. 38.2% of all prescriptions were issued for problems of the spine (“WS”), 35.0% for problems of the extremities (“EX”), and 6.5% for problems with the central nervous system (“ZN”). These prescriptions contained 242 procedure codes according to EBM. The most common chapter was “05: Normal physical therapy, one-on-one”, with 31.9%, “12: Manual therapy”, with 18.2% and “01: Medical massages”, with 9.9% of all prescribed procedure code. A total of 12.8% of the prescribed therapeutic remedies were to be performed in a house call. The estimated one-year prevalence to receive a prescription of this kind at least once was 5.7% [4.6–7.1%].

#### Medical aid products

A total of 381 prescriptions for medical aid products were billed for the study population. The most frequent chapters were “08: Shoe inlays”, with 16.8% of all prescriptions, “03: Application aides,” with 15% and “15: Incontinence aides”, with 10.8% of all prescriptions. Per 100 person-years, 21.4 prescriptions were billed (male: 20.9; female: 22.7). The estimate to receive a prescription of a medical aid product at least once in one year was 5.7% [4.6–7.0%].

#### Miscellaneous

The 517 billing documents regarding regular and emergency inpatient care will be analysed in a separate article.

The 934 remaining bills were caused by emergency and other transport services (*n* = 729), translator services (*n* = 28), home care (*n* = 82), midwifery bills (*n* = 40), statutory screening for new-borns (*n* = 30), inpatient rehabilitation (*n* = 3) and others/unknown (*n* = 22).

### Cost analyses

**Fig. 3:**
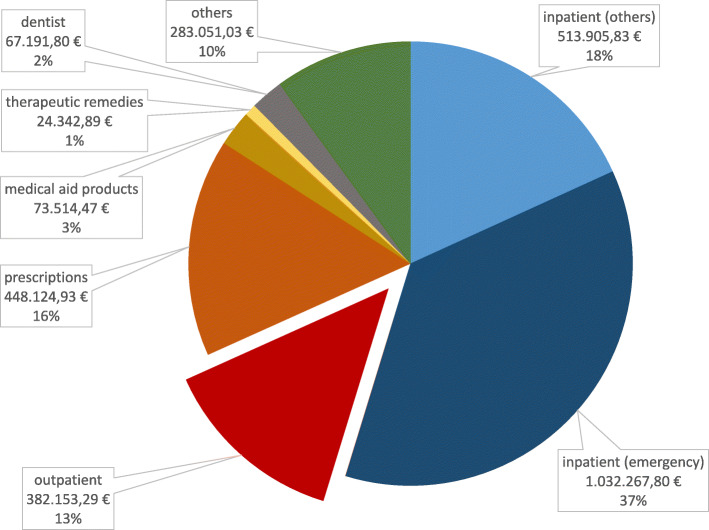
Proportions of costs per sector

In 2015, the social services office in Halle (Saale), Germany paid 2,825,106.52 € for medical care for asylum seekers. Per observed person-year, this amounted to 1584.33€ (men: 1178.39€; women: 2700.05€). This amount was lowest for children of 5 to 9 years (779€ per person-year) and highest for asylum seekers above the age of 55 years (3377 €). For this analysis, one extreme outlier was excluded: a premature baby who accounted for costs of more than 280,000€ alone. Figure 3 shows the total health care costs per sector of care. Inpatient care in total amounted to more than half (54.7%) of all costs, and inpatient care reported as emergency admissions resulted in 37% of all costs. Costs for the subgroup “others” were costs for emergency and other transport (59.9%), rehabilitation (13.9%), home care (13.7%), midwifery costs (4.5%), translator costs (3.8%) and miscellaneous/unknown (4.1%).

## Discussion

This study intended to describe health care utilization by asylum seekers and to identify characteristics in their utilization. To describe those in detail is an important first step to better understand how asylum seekers’ particular social and legal situation might affect their access to health care and, ultimately, their health. We identified four key characteristics that warrant further discussion: a low outpatient care utilization, the gender gap in most diagnoses and services, a large share of painkillers among all prescribed drugs and the total health care costs.

### Low outpatient care utilization

Public health researchers have long highlighted the high importance of early primary and outpatient care in limiting the later burden of disease in general [53] and for asylum seekers and other vulnerable groups in particular [22, 54]. Accordingly, it has been shown that a strong focus on primary health care for asylum seekers reduces health care costs to the public [55]. Access to health care for asylum seekers is complicated by structural factors such as language barriers or limited knowledge about the health system. In addition, with the exclusion of asylum seekers from the statutory health insurance through the ASBA and its various translations into practice, German health care policy has created artificial barriers in the access to health care for asylum seekers [10, 19, 37]. We suspect effects of these barriers in the utilization of health care services by our study population: When comparing our cohort of asylum seekers to the regularly insured German population [51], we found similar patterns in age and gender distribution contrasting consistently lower numbers of utilization of outpatient care across all age and gender groups. While among the regularly insured German population, more than 90% of all people visit an outpatient physician at least once in one year [51], at 67.5%, this prevalence was comparably low in our population. This might be assumed to show the “healthy migrant effect” [56], but a closer look reveals a more exact picture.

In detail, utilization of outpatient specialist care was lower than for the regularly insured population in every specialty [51], except for contacts to emergency departments [57]. Researchers have found high utilization of emergency care to be a result of inefficient and delayed ambulatory care and barriers in access to regular primary care [54, 58]. In emergency departments, as opposed to other outpatient offices, organizational barriers are low, as asylum seekers do not need health care vouchers there to receive treatment [59]. Limited knowledge about the structure of the German health system, with its separate inpatient and outpatient sectors and family doctors as designated first contact points for patients, also might lead asylum seekers to seek help in emergency departments first [58, 60, 61].

Despite many researchers reporting a high need for psychological care among refugees [11, 62–64], the prevalences of psychological diagnoses in our sample were low, and the proportion of people having contact with psychotherapists was extremely low. This might be explained by barriers in accessing psychological services and problems with reimbursement of psychotherapy by social security offices [62, 65]. In another publication, we expand on this topic by contrasting utilization data with data on actual health care needs in the same population to quantify this mismatch [66].

### Gender gap

With almost all of the measurements of utilization that we looked at, we found a large gender gap, with women having higher rates of outpatient cases and proportionally more women having contact with physicians, being diagnosed more often and receiving more prescriptions. This can only in part be explained through pregnancies and birth, as it holds true for almost all diagnosis groups and rates of health care services. These findings replicate other reports [21, 67, 68]. This might point towards lower access barriers for migrant women or higher health care needs, but we found no published research regarding this topic. More research is needed here to identify gender-specific modes of access and patterns of health care utilization among this population.

### Medication

Among the prescribed drugs for our study population, we found a surprisingly large percentage of common pain killers together making up more than 20% of all drug prescriptions made out for our study population. More than one-third of our population received at least one prescription for Ibuprofen in one year, compared to approximately one-fifth of the regularly insured population [69]. Similar results have been reported by Kahl and Frewer [70] from a sample in a reception centre in Bavaria. As they have, we can only speculate about reasons for this anomaly. Diagnoses of disorders of the musculoskeletal system and of symptoms of nonspecific pain were prevalent.

Nonspecific pain is a symptom often reported among asylum seekers [10, 63]. This might result from pain being a frequent symptom of psychological and psychosomatic conditions [71]. Previous studies have shown a high unmet need of treatment of such conditions and a tendency among mentally ill asylum seekers to report with somatic symptoms [72]. Another explanation might lie in the wording of the ASBA, stating in its first paragraph that asylum seekers are entitled to treatment for “acute and painful conditions” [17], which might lead physicians towards treating symptomatically with pain medication, especially when in uncertainty about actual entitlement regulations for their patients [24] or when unwilling or unable to engage for their patients to facilitate equitable treatment and to overcome barriers in access to adequate health care [26, 37]. Researchers working on asylum seekers’ perceptions of the health care system have described frequent pain killer prescriptions as a symbol for “*the lack of interest of and the rejection by the health care system”* [73], as they found pain killer prescription being a common trait among failed doctor-patient interactions with asylum seekers [37].

### Cost analyses

With regard to cost analyses, our findings for total cost per person-year are similar to findings by other researchers [18, 22, 65] in being much lower than for the regularly insured population in Germany, where the yearly total health expenditures per person in 2015 was reported to be 3019€ [74]. Bozorgmehr et al. [22] reported the total health care cost per person-year for asylum seekers in the German mean to be 1606€ in 2013. Bauhoff et al. [65] looked at a similar data source of a similar cohort of asylum seekers in 2016 who had access not through health care vouchers but who were handed an Electronic Health Insurance Card (EHIC), considerably facilitating access to health care [2]. With 1534 €, the total annual health care costs per person-year for our cohort were almost 20% lower than for Bauhoff et al.’s cohort of asylum seekers, at 1884€ [65]. Herein, the biggest relative differences are found in outpatient care and dentist costs. These are the sectors where organizational barriers in access are highest due to the local implementation of the ASBA and that were less cost-intensive in our cohort.

While this seems to support the rationale in restricting access to health care for asylum seekers to minimize costs to be carried by the German state [75], we are wary of this interpretation for reasons of ethics and economics. First, saving money by artificially creating an under-provision seems to be unethical to us and many others. Second, many researchers have argued that inadequate provision of primary and preventative care before [8], during [6] and after the flight [2, 10, 14, 59] would cause a shift in health services to later, more severe stages of disease and thereby to emergency care and the inpatient sector, ultimately making health care provision to this population more costly in the long term [2, 19, 22, 59]. We believe our data show signs of this shift in the analysis of costs per sector of care: more than half of all costs for our study population were generated in the inpatient sector compared to figures for the regularly insured, ranging from 25 to 39% [65, 74]. We also see a high share of costs generated through emergency hospitalization. This is in line with former research, showing high prevalences of hospitalization [67, 76] and high costs of inpatient health-care [54, 65] among asylum seekers. To reverse this shift back to the cost-efficient arena of primary and preventative care, this calls for initiatives to provide timely and adequate care both in the receiving country and along the migration routes to reduce the morbidity and health care costs in the long term [22, 28]. Further research is needed to investigate the causes and extent of preventable hospitalization among asylum seekers through restricted access to health care. We intend to give a more in-depth analysis of the data concerning regular and emergency inpatient care in a future publication.

### Strengths and limitations

Asylum seekers’ access to health care has been a controversial topic for many years. Nevertheless, scientific studies on the health-related effects of restricting access to health care have been scarce until now. With this article, we want to provide a first insight into health care utilization under circumstances of restricted access to health care structured through the ASBA and the provision of health vouchers to define a starting point for further analyses. As a major strength of our study, we consider that by using claims data from a social services office, we chose an approach that has thus far not been employed to generate data on asylum seekers’ health. With this population-based approach, we can show frequencies, prevalences and other key characteristics of the utilization of health care on an individual level, unrestrained by selection or recall bias. Our findings point to certain unmet health care needs among asylum seekers and can provide a baseline, to which similar data from different districts with different structural characteristics or from different time spans or data taken from other populations can be compared to evaluate measures to improve health outcomes among this vulnerable population.

However, the presented data alone do not allow for inferences about reasons for anomalies or characteristic patterns in health care utilization. The utilization of health care services is not congruent with the actual health care needs [72] and differs under differently structured modes of access [19]. Therefore, our data have to be interpreted against the backdrop of the local policies and conditions. In another publication, we contrast utilization data with data on actual health care needs, highlighting this difference between health care needs and utilization [66].

Our study population matched the general demographics of people coming to Germany as asylum seekers during that time in age and gender distribution, with young and male asylum seekers dominating the sample [77]. With regard to origin countries, similar to the national statistics, Syrian and Afghan nationals also dominated our study population. However, all nationalities from the Balkan states (Albania, Kosovo, Serbia) were largely missing from our sample. These countries are considered “safe origin countries” by the German government, and applicants from these countries are usually not distributed to the districts but have to stay in the receptions centres until their deportation. In turn, West African nationalities (Somalia, Benin, Guinea-Bissau, Niger) were overrepresented. Applicants from these countries had the longest durations of entitlement, i.e., asylum processes, and may thusly be overrepresented. Asylum seekers are distributed into the regions in Germany based on the “Königsteiner Schlüssel”, an allocation formula that calculates the number of asylum seekers to be taken in by each region according to its tax yield and its population count. While we know from informal discussions that the distribution of asylum seekers to the different federal states is additionally influenced by their nationality (with less-common nationalities being clustered in some federal states), we were not able to officially receive confirmation of these processes from the authorities in charge. Nevertheless, we do not assume that there is a strong association between nationality and health status and therefore consider the potential for bias arising from this problem to be rather small. The gender and age distribution, with a focus on male and younger participants, limits comparability to the population of the regularly insured in Germany.

Furthermore, interpretation of secondary data is limited by its origin [78]. Claims data are generated not for the purpose of research, but for billing of medical services, and therefore might be biased through the financial interest of service providers. Diagnosis codes on provider bills have to be noted to legitimate billed procedures and services; thus, diagnosis numbers may overestimate the true prevalence [51, 52]. However, our data on prescriptions only contains those documents that were turned in to pharmacies, but not those that were prescribed but never turned in. These data therefore only describe the drugs that were actually handed out to the asylum seekers in pharmacies. The data also do not contain information on prescription-free drugs and drugs that were given out as part of a hospital stay; thus, our figures may misrepresent true drug use to some extent [52].

Because of the structure of the data, we could not calculate certain characteristics that we had deemed important. We could not calculate the exact number of contacts between asylum seekers and physicians, as the billed cases can contain a number of contacts between care provider and patient. When looking at diagnoses we also could not differentiate between ongoing cases of one illness and multiple recurrences of the same disease so as to show incidences. This is a common problem also faced by other researchers using similar claims data [52, 69]. We drew on their work and also calculated one-year prevalences of diagnoses and utilization of different services to be able to compare our findings and to not underestimate the true figures.

## Conclusion

Empirical data on health care utilization by asylum seekers is essential to improve provision processes and outcomes. As of yet, German policy decisions on the health care of asylum seekers have not followed medical reasoning or empiric evidence and, thus, have had effects adverse to asylum seekers’ health. Germany has both accepted access to health care [79] as a human right and its implementation as its’ duty under EU law [80]. This means providing accessible, acceptable and high-quality health care to all those who need it is not only a moral obligation, but a legal one. As health professionals, we want to support the process towards health equity by contributing to a knowledge base about asylum seekers’ health care utilization. With this analysis of claims data held by the municipal social services office, we presented an exploration of a novel data source for monitoring utilization of health care by asylum seekers. With four characteristics in patterns of health care utilization, we identified fields of interest for further research: low outpatient care utilization, a substantial gender gap in utilization of almost all services and diagnosis groups, high shares of pain medication in drug prescriptions and a high share of costs being generated in the inpatient sector. We created a baseline, to which data from different districts of populations can be compared to. Further research regarding utilization under differently structured modes of access to health care and individual perceptions of the health system and barriers, as well as actual health care needs of asylum seekers, is needed to identify reasons for these characteristics and to deduce evidence-based measures to improve health care provision to asylum seekers.

## Supplementary information

**Additional file 1.** Additional information on the asylum process in Germany.

**Additional file 2.** Exemplary Kaplan-Meier analysis illustrating the method used in obtaining one-year prevalences.

**Additional file 3.** Additional data on prevalences by single diagnoses and diagnosis groups.

**Additional file 4.** Additional data on the Top 10 most prescribed single drugs.

## Data Availability

The data that support the findings of this study were obtained from the social services office, City of Halle (Saale), Germany (address: Fachbereich Soziales, Südpromenade 30, 06128 Halle (Saale), Germany), but restrictions apply to the availability of these data, which were used under license for the current study and therefore are not publicly available. Data are, however, available from the authors upon reasonable request and with written permission of the social services office, City of Halle (Saale), Germany.

## Abbreviations

CI: Confidence interval
ASBA: Asylum seekers benefits act (“*AsylbLG*”, “*Asylbewerberleistungsgesetz*”)
EBM: Uniform valuation scale for ambulatory physicians (“*Einheitlicher Bewertungsmaßstab*”)
BEMA: Valuation scale of dentistry services (“*Bewertungsmaßstab zahnärztlicher Leistungen*”)
ICD-10: International Classification of Diseases and Related Health Problems, 10th revision
PZN: Central pharmaceutical number (“*Pharmazentralnummer*”)
ATC: Anatomical Therapeutic Chemical Classification System
PY: Person-year
HIV: Humane immunodeficiency virus
EHIC: Electronic health insurance card
ZASt: Central reception centre for asylum seekers (“*Zentrale Anlaufstelle für Asylbewerber*”)
