# Supplementary material for: Outpatient health care utilization and health expenditures of asylum seekers in Halle (Saale), Germany - an analysis of claims data
Source: BMC Health Serv Res. 2020 Oct 20;20:961. doi: 10.1186/s12913-020-05811-4 (PMC7576695; doi:10.1186/s12913-020-05811-4)
Supplement: Supplementary file 1 — Additional file 1. Additional information on the asylum process in Germany. [file 12913_2020_5811_MOESM1_ESM.docx]

**Supplement 1:** The asylum process

Asylum seekers were mainly entering into our observation after their transfer to Halle (Saale) from the central reception centre for Saxony-Anhalt (ZASt) in Halberstadt or its branch offices, where the interview initiating the asylum process is held and a health screening is performed. In 2015, transfer to the districts of Saxony-Anhalt usually occured after 2-3 weeks (1). After the transfer to Halle (Saale), the asylum seekers were registered with the municipal social services office, which was the inclusion criterion for our study population. Asylum seekers exited our observation when they were deported after a negative decision regarding their request for asylum or when their request was approved, changing the asylum seeker’s legal status to refugee, which is connected with the right to have statutory health insurance (as in the general population). If the process regarding the request for asylum lasted longer than 15 months, there was also an option to enter the statutory health insurance if the authorities were not under the impression that the asylum seeker interfered with his or her asylum process. The asylum seekers are entitled only to provisions under the ASBA during this whole process.

Literature Cited

1. Aufenthaltszeit in zentralen Aufnahmeeinrichtungen für Geflüchtete in Sachsen-Anhalt: Drucksache 7/1400 Antwort der Landesregierung auf eine Kleine Anfrage zur schriftlichen Beantwortung Kleine Anfrage - KA 7/780; 2017 May 12.
