# Supplementary material for: Outpatient health care utilization and health expenditures of asylum seekers in Halle (Saale), Germany - an analysis of claims data
Source: BMC Health Serv Res. 2020 Oct 20;20:961. doi: 10.1186/s12913-020-05811-4 (PMC7576695; doi:10.1186/s12913-020-05811-4)
Supplement: Supplementary file 2 — Additional file 2. Exemplary Kaplan-Meier analysis illustrating the method used in obtaining one-year prevalences. [file 12913_2020_5811_MOESM2_ESM.docx]

**Supplement 2:** Exemplary Kaplan-Meier Analysis


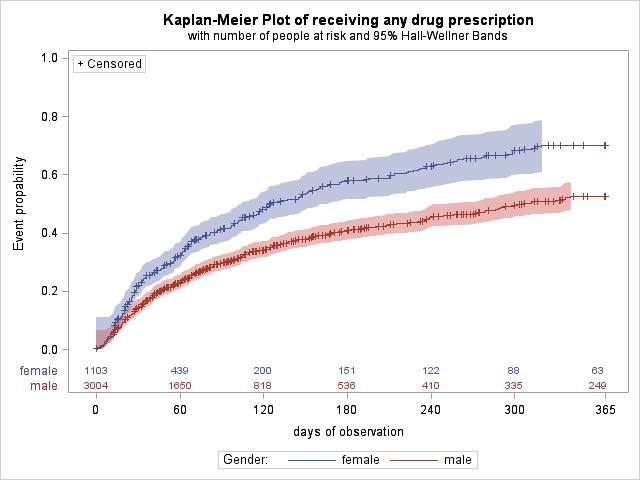


With failure-time analysis using the Kaplan-Meier method, we accounted for the fact that some asylum seekers entered our observation not on January 1^st^, 2015 or left the observation earlier than December 31, 2015. For each individual, the days are counted from the beginning of the observation until the event to be analysed, registered as a “failure”. We censored people leaving our observation without any registered event.

One-year estimates were taken from the last registered event during the observation time. In this example this occured after 340 days for men, with 52.6% [95%-CI: 49.7% - 55.6%] and after 319 days for women, with 70.0% [65.5% - 74.4%].
