# Supplementary material for: Outpatient health care utilization and health expenditures of asylum seekers in Halle (Saale), Germany - an analysis of claims data
Source: BMC Health Serv Res. 2020 Oct 20;20:961. doi: 10.1186/s12913-020-05811-4 (PMC7576695; doi:10.1186/s12913-020-05811-4)
Supplement: Supplementary file 3 — Additional file 3. Additional data on prevalences by single diagnoses and diagnosis groups. [file 12913_2020_5811_MOESM3_ESM.docx]

**Supplement 3:** Prevalences from outpatient care

Table 1: Most common diagnosis codes (ICD-10), with estimated one-year prevalences of people being diagnosed at least once

| Diagnosis | | | | | | | | | One-year prevalence | |  | |  | | |
| --- | --- | --- | --- | --- | --- | --- | --- | --- | --- | --- | --- | --- | --- | --- | --- |
| Codes | | | | | | | **Description** | | **% of female (95%-CI)** | | **% of all (95%-CI)** | | **% of all (95%-CI)** | | |
| J06 | | | | | | | Acute upper respiratory infections of multiple and unspecified sites | | 13.6 (11.8 - 15.7) | | 23.2 (19.5 - 27.4) | | 16.1 (14.5 - 18.0) | | |
| R10 | | | | | | | Abdominal and pelvic pain | | 10.7 (9.0 - 12.6) | | 29.1 (25.2 - 33.5) | | 15.6 (13.9 - 17.4) | | |
| M54 | | | | | | | Dorsalgia | | 13.1 (11.3 - 15.2) | | 15.8 (12.8 - 19.5) | | 13.8 (12.2 - 15.5) | | |
| Z27 | | | | | | | Need for immunization against combinations of infectious diseases | | 5.7 (4.6 - 7.1) | | 11.9 (9.3 - 15.2) | | 7.4 (6.2 - 8.7) | | |
| K29 | | | | | | | Gastritis and duodenitis | | 7.0 (5.6 - 8.7) | | 8.6 (6.4 - 11.5) | | 7.4 (6.2 - 8.8) | | |
| F43 | | | | | | | Reaction to severe stress, and adjustment disorders | | 4.6 (3.5 - 6.0) | | 8.3 (6.3 - 11.0) | | 5.5 (4.5 - 6.7) | | |
| L30 | | | | | | | Other dermatitis | | 5.2 (4.1 - 6.6) | | 7.5 (5.4 - 10.5) | | 5.9 (4.8 - 7.1) | | |
| R51 | | | | | | | Headache | | 5.3 (4.2 - 6.7) | | 8.3 (6.1 - 11.3) | | 6.1 (5.0 - 7.3) | | |
| Z30 | | | | | | | Contraceptive management | | - | | 20.4 (16.9 - 24.6) | | 5.5 (4.4 - 6.7) | | |
| Z00 | | | | | | | General examination and investigation of persons without complaint and reported diagnosis | | 3.3 (2.5 - 4.4) | | 12.4 (9.8 - 15.6) | | 5.8 (4.8 - 6.9) | | |
| F32 | | | | | | | Depressive episode | | 3.3 (2.4 - 4.4) | | 6.0 (4.2 - 8.5) | | 4.0 (3.2 - 5.0) | | |
| N89 | | | | | | | Other noninflammatory disorders of vagina | | - | | 17.7 (14.5 - 21.6) | | 4.7 (3.8 - 5.9) | | |
| N39 | | | | | | | Other disorders of urinary system | | 2.2 (1.5 - 3.1) | | 11.0 (8.4 - 14.3) | | 4.6 (3.6 - 5.7) | | |
| Table 2: Morbidity by selected diagnosis groups, with estimated one-year prevalence of people being diagnosed at least once | | | | | | | | | | | | | | | |
| Diagnoses | | | |  | |  | |  | | **One-year prevalence** | | | |  |  |
| Codes | |  | |  | **Description** | | | | | **% of male (95%-CI)** | | **% of female (95%-CI)** | | **% of all (95%-CI)** |  |
| A00 | - | | A09 | | Intestinal infectious diseases | | | | | 3.3 (2.4 - 4.5) | | 7.7 (5.5 - 10.7) | | 4.5 (3.6 - 5.6) |  |
| A50 | - | | A64 | | Infections with a predominantly sexual mode of transmission | | | | | 0.9 (0.5 - 1.5) | | 0.7 (0.2 - 2.6) | | 0.8 (0.5 - 1.4) |  |
| B15 | - | | B19 | | Viral hepatitis | | | | | 1.8 (1.1 - 2.8) | | 0.5 (0.2 - 1.7) | | 1.4 (0.9 - 2.2) |  |
| B65 | - | | B83 | | Helminthiases | | | | | 1.2 (0.7 - 2.0) | | 1.2 (0.5 - 2.9) | | 1.2 (0.8 - 1.9) |  |
| B85 | - | | B89 | | Pediculosis, acariasis and other infestations | | | | | 3.1 (2.3 - 4.2) | | 2.5 (1.4 - 4.5) | | 3.0 (2.3 - 3.9) |  |
| B95 | - | | B98 | | Bacterial, viral and other infectious agents | | | | | 1.0 (0.6 - 1.8) | | 2.0 (1.0 - 3.9) | | 1.3 (0.8 - 2.0) |  |
| D50 | - | | D53 | | Nutritional anaemias | | | | | 0.7 (0.4 - 1.3) | | 7.5 (5.4 - 10.3) | | 2.5 (1.9 - 3.4) |  |
| E10 | - | | E14 | | Diabetes mellitus | | | | | 1.0 (0.6 - 1.7) | | 2.0 (1.1 - 3.5) | | 1.3 (0.9 - 1.9) |  |
| E40 | - | | E46 | | Malnutrition | | | | | 0 | | 0 | | 0 |  |
| E65 | - | | E68 | | Obesity and other hyperalimentation | | | | | 0.4 (0.2 - 0.8) | | 3.2 (2.1 - 4.9) | | 1.2 (0.8 - 1.7) |  |
| I10 | - | | I15 | | Hypertensive diseases | | | | | 2.5 (1.8 - 3.5) | | 4.8 (3.3 - 7.1) | | 3.1 (2.4 - 4.0) |  |
| I20 | - | | I25 | | Ischaemic heart diseases | | | | | 0.9 (0.6 - 1.5) | | 2.8 (1.6 - 4.9) | | 1.5 (1.0 - 2.1) |  |
| I60 | - | | I69 | | Cerebrovascular diseases | | | | | 0.4 (0.1 - 1.2) | | 0.7 (0.2 - 2.1) | | 0.5 (0.2 - 1.0) |  |
| J09 | - | | J18 | | Influenza and pneumonia | | | | | 1.2 (0.8 - 2.0) | | 2.5 (1.4 - 4.4) | | 1.6 (1.1 - 2.3) |  |
| J40 | - | | J47 | | Chronic lower respiratory diseases | | | | | 2.8 (2.0 - 4.0) | | 6.4 (4.5 - 8.9) | | 3.8 (3.0 - 4.8) |  |
| N80 | - | | N98 | | Noninflammatory disorders of female genital tract | | | | | 0 | | 32.4 (28.2 - 37.0) | | 8.5 (7.2 - 9.9) |  |
| O00 | - | | O08 | | Pregnancy with abortive outcome | | | | | 0 | | 3.1 (1.9 - 5.1) | | 0.8 (0.5 - 1.4) |  |
| O09 | - | | O09 | | Length of pregnancy | | | | | 0 | | 10.4 (7.9 - 13.7) | | 2.8 (2.1 - 3.8) |  |
| O10 | - | | O16 | | Oedema, proteinuria and hypertensive disorders in pregnancy, childbirth and the puerperium | | | | | 0 | | 1.3 (0.5 - 2.9) | | 0.4 (0.1 - 0.8) |  |
| O20 | - | | O29 | | Other maternal disorders predominantly related to pregnancy | | | | | 0 | | 10.4 (7.9 - 13.6) | | 2.8 (2.1 - 3.8) |  |
| O30 | - | | O48 | | Maternal care related to the foetus and amniotic cavity and possible delivery problems | | | | | 0 | | 10.0 (7.6 - 13.1) | | 2.7 (2.0 - 3.6) |  |
| O60 | - | | O75 | | Complications of labour and delivery | | | | | 0 | | 2.1 (1.1 - 4.0) | | 0.6 (0.3 - 1.1) |  |
| O80 | - | | O82 | | Delivery | | | | | 0 | | 1.9 (0.9 - 3.9) | | 0.5 (0.2 - 1.1) |  |
| O85 | - | | O92 | | Complications predominantly related to the puerperium | | | | | 0 | | 1.8 (0.9 - 3.8) | | 0.5 (0.2 - 1.1) |  |
| O94 | - | | O99 | | Other obstetric conditions, not elsewhere classified | | | | | 0 | | 6.2 (4.4 - 8.7) | | 1.7 (1.2 - 2.4) |  |
| P05 | - | | P08 | | Disorders related to length of gestation and foetal growth | | | | | 0 | | 0.1 (0.0 - 0.6) | | 0.0 (0.0 - 0.2) |  |
| X60 | - | | X84 | | Intentional self-harm | | | | | 0.1 (0.0 - 0.9) | | 0 | | 0.1 (0.0 - 0.7) |  |
| Z20 | - | | Z29 | | Persons with potential health hazards related to communicable diseases | | | | | 8.3 (6.9 - 10.0) | | 16.7 (13.6 - 20.4) | | 10.6 (9.2 - 12.1) |  |
| Z55 | - | | Z65 | | Persons with potential health hazards related to socioeconomic and psychosocial circumstances | | | | | 0.1 (0.0 - 0.5) | | 1.6 (0.8 - 3.3) | | 0.5 (0.3 - 1.0) |  |
