# Supplementary material for: Outpatient health care utilization and health expenditures of asylum seekers in Halle (Saale), Germany - an analysis of claims data
Source: BMC Health Serv Res. 2020 Oct 20;20:961. doi: 10.1186/s12913-020-05811-4 (PMC7576695; doi:10.1186/s12913-020-05811-4)
Supplement: Supplementary file 4 — Additional file 4. Additional data on the Top 10 most prescribed single drugs. [file 12913_2020_5811_MOESM4_ESM.docx]

**Supplement 4:** Top 10 of the most prescribed drugs with percentages of all prescriptions and one-year prevalences of people receiving at least one prescription (stratified for gender)

| Prescribed drugs | | Proportion | One-year prevalence | |  |
| --- | --- | --- | --- | --- | --- |
| Code | **Drug name** | **% of all prescriptions** | **% of male (95%-CI)** | **% of female (95%-CI)** | **% of all (95%-CI)** |
| M01AE01 | Ibuprofen | 14.1 | 31.0 (28.4 - 33.9) | 39.7 (35.1 - 44.6) | 33.4 (31.0 - 35.8) |
| A02BC02 | Pantoprazole | 5.2 | 13.4 (11.5 - 15.5) | 14.1 (11.3 - 17.6) | 13.5 (11.9 - 15.3) |
| R01AA07 | Xylometazoline | 4.1 | 4.8 (3.8 - 6.2) | 11.7 (9.2 - 14.9) | 6.6 (5.5 - 7.9) |
| N02BB02 | Metamizole sodium | 3.9 | 10.5 (8.8 - 12.4) | 15.1 (11.9 - 19.0) | 11.8 (10.2 - 13.5) |
| N02BE01 | Paracetamol | 1.7 | 3.3 (2.4 - 4.6) | 9.6 (7.2 - 12.7) | 5.0 (4.0 - 6.2) |
| N06AX11 | Mirtazapine | 1.6 | 1.7 (1.1 - 2.6) | 3.2 (1.9 - 5.3) | 2.1 (1.5 - 2.9) |
| M01AB05 | Diclofenac | 1.3 | 3.8 (2.9 - 5.1) | 3.3 (2.0 - 5.5) | 3.7 (2.9 - 4.8) |
| R05CP02 | Hederae helicis folium | 1.3 | 1.8 (1.2 - 2.6) | 6.6 (4.6 - 9.6) | 3.2 (2.4 - 4.2) |
| H03AA01 | Levothyroxine sodium | 1.1 | 0.6 (0.3 - 1.2) | 4.5 (3.0 - 6.8) | 1.6 (1.1 - 2.3) |
| V04CA03 | Glucose test, blood | 1.0 | 0.4 (0.1 - 1.0) | 1.6 (0.7 - 3.6) | 0.7 (0.4 - 1.3) |
